# Use of a Two-Eyed Seeing Approach to Increase Client Satisfaction with Psychiatric Services Among an Indigenous Population

**DOI:** 10.1192/j.eurpsy.2023.744

**Published:** 2023-07-19

**Authors:** L. Mehl-Madrona, B. Mainguy

**Affiliations:** 1 Clinical Services, Wabanaki Public Health and Wellness, Bangor; 2 Intermedia, University of Maine, Orono, United States

## Abstract

**Introduction:**

Psychiatry has historically had difficult relationships with Indigenous people, tending to dismiss their views on mental health and illness as superstitious or primitive. Indigenous people have responded by refusing to come for services, missing appointments, and dismissing treatment offered. Canadian Mi’qmak elder Albert Marshall introduced the term, *etuaptmumk,* to highlight the idea that Indigenous Knowledge and Practice has equal value to contemporary psychiatry. While translated as two-eyed seeing, the term refers to explanatory pluralism and invites a respectful dialogue among cultures as equals.

**Objectives:**

We implemented a two-eyed seeing research in Indigenous communities to explore the differences of some Indigenous North American cultures in their view of mind and mental health from conventional Euro-American psychiatry and how their cultural practitioners negotiated those differences. We wondered how perception of services would change if mental health practitioners were aware and were more respectful of those differences.

**Methods:**

We engaged in a series of discussions with traditional knowledge keepers about their views on mind and mental health. We used constructivist grounded theory methods to identify what was common among these views. We developed a training program to engage mental health clinicians in understanding and responding to these differences. We engaged community members receiving services in a discussion about their perception in the change in the quality of the services. We summarized their responses.

**Results:**

Differences between Indigenous views and those of conventional psychiatry included (1) greater emphasis on the role of community and socio-environmental influences on mental health, (2) inclusion of spirituality and spiritual beings in their expectation for cause of problems and treatment, (3) greater emphasis on inter-generational trauma and historical trauma in addressing mental health. After inclusion of practitioners in the mental health services who had been oriented to these differences and integration of ceremony into the mental health services, ninety-four percent of respondents asked if the service had improved responded in the positive. Attendance at scheduled appointments increased from 56% to 81%. Adherence to recommendations increased from 27% to 67%, which were significant.

**Conclusions:**

Awareness of cultural differences in beliefs about causes of mental illness and acceptable types of treatment increased client satisfaction with services offered, improved attendance rates, and improved adherence rates to treatments offered among Indigenous people. These results could generalize to other populations who hold different views than those of conventional psychiatry. The concept of two-eyed seeing, or explanatory pluralism, holds heuristic value for psychiatry by allowing multiple points of view to be true at the same time.

**Disclosure of Interest:**

None Declared

